# Interaction and Redox Chemistry between Iron, Dopamine, and Alpha-Synuclein C-Terminal Peptides

**DOI:** 10.3390/antiox12040791

**Published:** 2023-03-24

**Authors:** Fabio Schifano, Simone Dell’Acqua, Stefania Nicolis, Luigi Casella, Enrico Monzani

**Affiliations:** 1 Department of Chemistry, University of Pavia, Via Taramelli 12, 27100 Pavia, Italy; 2 IUSS School for Advanced Studies of Pavia, Palazzo del Broletto, Piazza della Vittoria 15, 27100 Pavia, Italy

**Keywords:** α-synuclein, iron, dopamine, Parkinson’s disease, oxidative stress

## Abstract

α-Synuclein (αS), dopamine (DA), and iron have a crucial role in the etiology of Parkinson’s disease. The present study aims to investigate the interplay between these factors by analyzing the DA/iron interaction and how it is affected by the presence of the C-terminal fragment of αS (Ac-αS_119–132_) that represents the iron-binding domain. At high DA:Fe molar ratios, the formation of the [Fe^III^(DA)_2_]^–^ complex prevents the interaction with αS peptides, whereas, at lower DA:Fe molar ratios, the peptide is able to compete with one of the two coordinated DA molecules. This interaction is also confirmed by HPLC-MS analysis of the post-translational modifications of the peptide, where oxidized αS is observed through an inner-sphere mechanism. Moreover, the presence of phosphate groups in Ser129 (Ac-αS^p^S_119–132_) and both Ser129 and Tyr125 (Ac-αS^p^Y^p^S_119–132_) increases the affinity for iron(III) and decreases the DA oxidation rate, suggesting that this post-translational modification may assume a crucial role for the αS aggregation process. Finally, αS interaction with cellular membranes is another key aspect for αS physiology. Our data show that the presence of a membrane-like environment induced an enhanced peptide effect over both the DA oxidation and the [Fe^III^(DA)_2_]^–^ complex formation and decomposition.

## 1. Introduction

**Scheme 1:**
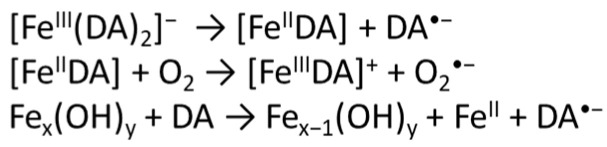
Iron-mediated DA oxidation occurs mainly by the [Fe^III^(DA)_2_]^−^ complex, through internal electron transfer from bound DA to Fe^III^, yielding Fe^II^ and DA semi-quinone (DA^•−^). The [Fe^II^-DA] complex is then readily oxidized by O_2_, producing superoxide anion [9]. Alternatively, amorphous aggregates of iron hydroxide promote DA oxidation with concomitant Fe^II^ release. Please note that when DA is complexed to iron(III), as in [Fe^III^DA]^+^, it is assumed that it binds in its dianionic form (DA^•−^).

The interplay between iron, dopamine (DA), and α-synuclein (αS) seems to be a key feature of degenerating neurons in Parkinson’s disease (PD) [1]. In fact, from one side, it is well known that iron is able to exacerbate DA toxicity by promoting its oxidation to neurotoxic species [2], while, on the other hand, αS is able to bind to iron in both oxidation states [3]. The presence of an intracellular pool of labile, chelatable iron has long been recognized [4], and recent progress toward its characterization and distribution has been made in bacterial cells [5]. More iron can be released by damaged neurons, particularly those of the *substantia nigra* (SN), which contain the iron-rich neuromelanin pigment [6]. The dopaminergic neurons of SN are, in fact, particularly vulnerable to oxidative stress, mainly due to their direct involvement in DA metabolism which, if not controlled, can lead to the production of reactive quinones and ROS [7]. Furthermore, the produced quinones are able to react with proteins, like αS, thus preventing the protein from exerting its physiological role and ultimately leading to a vicious cycle in which the increased cytosolic DA exacerbates the oxidative stress [8]. Iron, being a redox-active transition metal, can promote DA oxidation (Scheme 1) by overcoming the spin restriction nature of dioxygen reduction. In addition, the produced superoxide and hydrogen peroxide can be converted into hydroxyl radical through Fenton- and Haber–Weiss-type reactions. Alongside the quinones and ROS production, iron–DA interaction can produce 6-hydroxydopamine, a neurotoxin used to simulate PD in animal models, although to a modest extent [2].

The interaction between iron and DA is strongly dependent on the DA:Fe molar ratio; at low molar ratio the interaction is pro-oxidant, leading to the production of ROS and Fenton chemistry, while at high molar ratio, where the coordinatively saturated [Fe^III^(DA)_3_]^3–^ species is formed, the interaction is protective, since the strong DA complexation hinders the reaction between iron and hydrogen peroxide. Despite this, the internal electron transfer is still possible, leading to the same toxic DA quinones [9]. It is worth noting that DA is able to mobilize iron from amorphous aggregates of iron hydroxide through a reductive mechanism (Scheme 1). These latter aspects of Fe–DA redox chemistry are of paramount importance in promoting toxicity in the long term, also considering the boundary conditions observed in PD, like decreased ferritin levels, impaired iron homeostasis, and decreased DA levels as well [10].

Regarding the effects of αS-iron interaction, in addition to the ability of the protein to bind to the metal ion, the protein contains an iron-responsive element in its 5’-untranslated region, like ferritin; thus, the protein translation appears to be regulated by iron levels. In depleted iron conditions, protein synthesis is inhibited, while, in the case of iron overload, the protein is synthesized [3]. Nevertheless, metal binding makes the protein more prone to aggregation by stabilizing particular local conformations, reducing electrostatic repulsion, and even acting as a cross-linker [11]. Remarkably, iron overload could start a vicious toxic cycle in which the excess iron level promotes protein translation and, ultimately, the related toxicity.

**Figure 1:**
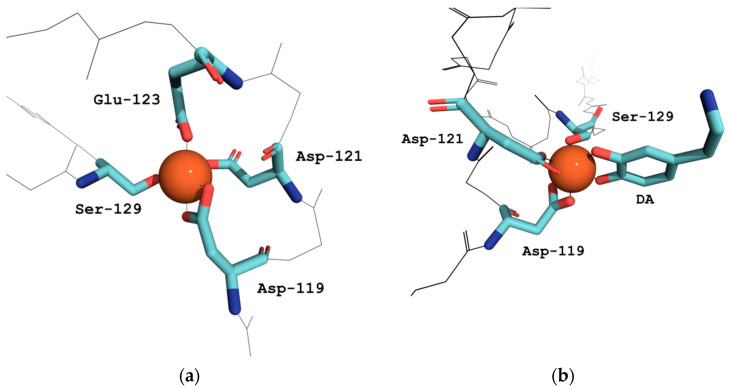
(**a**) Schematic representation of the putative iron coordination sphere of αS [12,13]. (**b**) Schematic representation of one of the possible [Fe(DA)(Ac-αS_119–132_)] ternary complexes, where Fe^III^ is bound to D119, D121, and S129. Both sketches were drawn using the Avogadro and PyMol softwares [17,18]; the peptide’s backbone was drawn as poly-Gly.

**Scheme 2:**
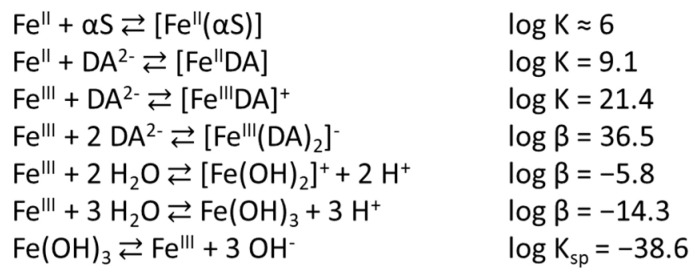
Reactions and related constants for Fe^II^-DA and Fe^III^-DA binding, Fe^III^ hydrolysis, and Fe(OH)_3_ solubility. All reported constants refer to 25 °C and infinite dilution [9,15,16].

The iron-binding site in αS is located in the C-terminus of the protein encompassing the ^119^DPDNEA^124^ motif, in which D119 acts as the main anchoring residue. The coordination sphere is poorly characterized, but it seems that D121, E123, and S129 participate in iron binding (Figure 1) [12,13]. Furthermore, precise binding location also relies on the phosphorylation state of the protein where, upon such modification, a shift in the coordination sphere is likely to occur [12,13]. Phosphorylation also enhances the iron-binding properties between 10 and 50-fold when compared to the wild type [13,14]. In spite of the appreciable protein affinity towards iron [15], in aerated solutions at circumneutral pH and in the presence of DA, iron hydrolysis and [Fe^III^(DA)_2_]^–^ complex formation overcomes iron binding to αS, as can be deducted from the log K values reported in Scheme 2 [9,16].

The reactivity of DA and iron ions has been extensively studied by Waite et al. in various conditions, and complex kinetic modeling was used to deduce rate constants for the various reactions involved [9,10]. The effect of iron binding by αS on this chemistry, however, is not known, and we aimed to investigate the reactivity of the Fe–DA system using the αS *C*-terminal peptide Ac-^119^DPDNEAYEMPSEEG^132^-NH_2_ (Ac-αS_119–132_), containing the iron-binding site and the phosphorylated mutant Ac-^119^DPDNEAYEMP(pS)EEG^132^-NH_2_ (Ac-αS^p^S_119–132_). The choice of this particular mutant was made based on the finding that phosphorylated αS at S129 is the major form present in Lewy bodies [19], and that phosphorylation increases the binding affinity of the protein for both iron(II) and iron(III) [14,20].

Furthermore, the phosphorylation of Y125 is indicated to be involved in the pathological mechanism of αS aggregation [21,22]; however, to date, the role of this post-translational modification has not been analyzed at the molecular level. For this reason, we decided to study the interaction between iron and αS also with the peptide bearing the phosphate group at both S129 and Y125 (Ac-^119^DPDNEA(pY)EMP(pS)EEG^132^-NH_2_ (Ac-αS^p^Y^p^S_119–132_).

Another important issue to consider is that, whereas αS is unstructured in solution, its interaction with lipid membrane modulates the conformation by inducing an α-helical structure of the *N*-terminal region [23]. This conformational change delays the oligomerization of the protein [24] and modulates the interaction with metal ions, as recently observed for copper-αS complex formation [25,26]. For this reason, the analysis of the interaction between iron and αS should also consider the presence of a membrane-like environment.

## 2. Materials and Methods

### 2.1. Materials

Protected amino acids, rink amide resin, and benzotriazole-1-yl-oxy-tris-pyrrolidino-phosphonium hexafluorophosphate (PyBOP) were purchased from Novabiochem, while all other reagents were purchased from Merck at the highest purity available and used as received. All glassware was acid-washed with 6 M HCl for 1 week and rinsed thoroughly with ultrapure water. All aqueous solutions were prepared in ultrapure 18 MΩ Milli-Q water. Stock solutions of Fe^II^ were prepared by dissolving FeSO_4_·7H_2_O in 10 mM H_2_SO_4_. Stock solutions of Cu(II) were made by dissolving CuSO_4_·5H_2_O in water. Stock solutions of dopamine were prepared in HCl 10 mM. Stock solutions of Ac-αS_119–132_, Ac-αS^p^S_119–132_, and Ac-αS^p^Y^p^S_119–132_ were prepared by dissolving the corresponding peptide in H_2_O; the concentrations were assessed by UV-Vis spectroscopy, measuring the absorbance of Y125 (λ_max_ = 275 nm, ε_275_ = 1420 M^−1^ cm^−1^) or pY125 (λ_max_ = 266 nm, ε_266_ = 500 M^−1^ cm^−1^) [20]. Stock solutions of horseradish peroxidase (HRP) were prepared by dissolving the enzyme in H_2_O, and the concentration was assessed by UV-Vis spectroscopy, measuring the absorbance of the Soret band of the protein (λ_max_ = 402 nm, ε_402_ = 103,000 M^−1^ cm^−1^). The buffers used were adjusted to proper pH using NaOH, H_3_PO_4_, HCl, or HNO_3_, depending on the buffer type.

### 2.2. Instrumentation

#### 2.2.1. RP-HPLC

The chromatographic separations were operated with a Shimadzu Prominence instrument equipped with a DGU-20A_3R_ degassing unit, two LC-20AD pumps, an SPD-M20A photodiode array (190–800 nm wavelength range), and a CTO-20A column oven. The columns used were a Phenomenex Jupiter 4U Proteo 90A, 250 × 10 mm as semi-preparative and a Phenomenex Jupiter 4U Proteo 90A, 250 × 4.6 mm or a Supelco Ascentis Express 2.7 µm C18, 100 × 4.6 mm as analytical. The solvents used were CH_3_CN and H_2_O both with 0.1% TFA for gradient elution or 98% HCOOH pH 2 plus 2% CH_3_CN for isocratic elution.

#### 2.2.2. Mass Spectrometry

The MS and MS/MS spectra were recorded with a Thermo Finnigan LCQ ADV MAX equipped with an ionic-trap and an ESI ion source. The spectra were analyzed with Xcalibur 2.0.7 SP1 software.

#### 2.2.3. HPLC-MS

The HPLC-MS analysis was performed on a Thermo Finnigan HPLC autosampler Surveyor IC pump coupled with a Thermo Finnigan LCQ ADV MAX. Fragmentation was achieved by collision-induced dissociation (CID), with an isolation width of 2 Th (*m*/*z*); the activation amplitude was around 35% of the ejection radio frequency amplitude of the instrument. The column used was a Phenomenex Jupiter 4U Proteo 90A, 150 × 2 mm, flow rate 0.2 mL/min. The solvents used were H_2_O plus 0.1% HCOOH (A) and CH_3_CN plus 0.1% HCOOH (B). Separations were performed with a semi-linear gradient (0–5 min 2% B, 5–50 min 2–33% B, 50–55 min 33–100% B, 55–73 min 100% B, 73–78 100–2% B, and 78–90 min 2% B). Data were analyzed with Xcalibur^TM^ 2.0.7 SP1 software.

#### 2.2.4. UV-Vis Spectroscopy

The UV-Vis spectra were acquired with an Agilent 8453 spectrophotometer, equipped with a 1024 photodiode array detector (190–1100 nm wavelength range) and a magnetically stirred, thermostated sample holder. All spectra were recorded in a quartz cell with an optical path of 1 cm.

### 2.3. General Methods

#### 2.3.1. Peptide Synthesis

The Ac-αS_119–132_ (Ac-^119^DPDNEAYEMPSEEG^132^-NH_2_, M.W. 1623.6 g/mol) was synthesized by standard 9-fluorenylmethoxycarbonyl (Fmoc) solid-phase peptide synthesis (SPPS) in DMF, using a 0.33 scale. In order to obtain the amidated C-terminus of the peptide, MBHA rink amide resin (100–200 mesh) was employed as solid support. Resin deprotection was achieved by treating the solid phase with 2 × 10 mL of 20% (*v*/*v*) piperidine in DMF for 3 and 7 mins. After this, the first amino acid was condensed by adding 2 equivalents (vs reactive sites) 1-hydroxybenzotriazole (HOBt), PyBOP and N,N-diisopropylethylamine (Dipea). Coupling time was set to 45 min and, after this, the resin was washed with 10 mL of DMF and the coupling procedure was repeated. Unreacted N-terminal groups were acetylated with 2 × 10 mL 4.7% acetic anhydride and 4% pyridine in DMF for 15 and 7 mins, respectively. Coupling yield was evaluated spectrophotometrically reading the absorbance of the dibenzofulvene–piperidine adduct (λ_max_ = 301 nm, ε_301_ = 7800 M^−1^ cm^−1^) generated upon the deprotection process. In order to have acetylated N-terminus of the peptide, after the last coupling, the peptide was first deprotected and then acetylated. Resin was dried by washing with 3 × 10 mL of DMF, DCM, 2-propanol, and Et_2_O. Cleavage of the peptide from the resin and deprotection of the side chains were achieved at once by treating the resin with 25 mL 95/2.5/2.5 (*v*/*v*) of TFA/H_2_O/triisopropylsilane, under stirring in the dark at r.t. for 3 h. Then, the resin was filtered out and the peptide was precipitated with 200 mL of cold Et_2_O, washed with Et_2_O, and filtered. The crude peptide was solubilized in H_2_O and purified by RP-HPLC (flow rate 5 mL/min, loop 2 mL, oven 25 °C, semi-preparative column) using a semi-linear gradient of 0.1% TFA in H_2_O (A) and 0.1% of TFA in CH_3_CN (B) (0–8 min 2% B, 8–40 min 2–55% B, and 40–50 min 55–100% B, retention time 16.5 min). Elution was monitored reading the absorbance at 200 and 280 nm. The purified peptide was lyophilized and stored at −20 °C. The product was characterized by ESI-MS (direct injection, MeOH, negative-ion mode, capillary temperature 200 °C): *m*/*z* 1622 ([M-H]^−^), 811 ([M-2H]^2−^). The purity was determined by HPLC-MS (>95%).

Ac-αS^p^S_119–132_ (Ac-^119^DPDNEAYEMP(pS)EEG^132^-NH_2_, M.W. 1703.6 g/mol) was synthesized and purified following the same protocol; phosphorylated amino acid was incorporated as monobenzyl-protected Fmoc-Ser(PO(OBzl)OH). RP-HPLC: retention time 17.4 min; ESI-MS: *m*/*z* 1702 ([M-H]^−^), 851 ([M-2H]^2−^); purity > 95%.

Ac-αS^p^Y^p^S_119–132_ (Ac-^119^DPDNEA(pY)EMP(pS)EEG^132^-NH_2_, M. W. 1783.6 g/mol) was synthesized and purified following the same protocol, but the elution was monitored reading the absorbance at 200 and 266 nm. Phosphorylated amino acids were incorporated as monobenzyl-protected Fmoc-Tyr(PO(OBzl)OH)OH and Fmoc-Ser(PO(OBzl)OH). RP-HPLC: retention time 18.8 min; ESI-MS: *m*/*z* 1782 ([M-H]^−^), 891 ([M-2H]^2−^); purity > 95%.

#### 2.3.2. Effect of αS-Peptides on Fe^III^-DA Interaction

The effect of peptides on Fe^III^-DA complex formation was studied at 37 °C in 10 mM Mops buffer pH 7.0 with 100 mM NaCl as the ionic strength buffer. The reactions were monitored by UV-Vis spectroscopy, following the absorption of the [Fe^III^(DA)_2_]^–^ complex (λ_max_ = 580 nm, ε_580_ = 3312 M^−1^ cm^−1^) for 150 s with 0.5 s shutter cycle. The kinetic traces were obtained by subtracting the absorbance at 900 nm to the absorbance of the [Fe^III^(DA)_2_]^–^ complex at 580 nm and plotting the results against time. The DA:Fe molar ratio was kept constant at 2:1, with 200 µM DA and 100 µM Fe^II^, while the selected peptide concentration was changed from 0 to 200 µM, with Fe^II^ being the last reagent added. The experiments with 20 mM SDS, as the membrane model, were carried out with 200 µM DA, 100 µM Fe^II^, and 200 µM of the selected peptide. The experiments were also repeated with a higher DA:Fe molar ratio of 20:1, changing the Fe^II^ concentration to 10 µM.

#### 2.3.3. Kinetic Studies on the Oxidation of DA Promoted by Fe^III^-Peptide Complexes

UV-Vis spectroscopy: The catalytic oxidation of DA promoted by Fe^III^-peptide complexes was studied at 37 °C in 10 mM Mops buffer pH 7.0 with 100 mM NaCl as the ionic strength buffer. The reactions were monitored by UV-Vis spectroscopy, following the absorption of DA (λ_max_ = 281 nm) for 24 h, recording spectra at 0, 1.5, 3, 6, and 24 h. The background contribution was removed with a three-point-dropline correction. Briefly: two reference wavelengths, which define the slope, were selected on either side of the analytical wavelength and used to extrapolate the background contribution at the analytical wavelength (i.e., 281 nm). The absorbance readings were plotted against time obtaining the kinetic traces. For DA autoxidation, in a plastic tube covered with tinfoil, DA was added to a final volume of 10 mL of buffer (DA final concentration 200 µM). An aliquot of 2 mL was then taken and used to record a UV-Vis spectrum (t = 0 h), and then unified with the bulk solution. The tube was covered with a holed sheet of tinfoil and left in a thermostatic bath at 37 °C. After 1.5 h, a new aliquot was taken, centrifuged (14,000 rpm, 4 min), analyzed, and unified again with the bulk solution. The latter procedure was repeated at the selected times. For evaluating the effect of Fe, after the recording of the first spectrum, Fe^II^ was added to the DA solution (final concentration 10 µM) and an aliquot of 2 mL was newly taken and used to record a second UV-Vis spectrum (t = 0 h), and then reunified with the bulk solution. Last, the effect of Fe^III^-peptide complexes was evaluated by adding 20 µM of selected peptide before adding the metal ion. The whole protocol was repeated adding 20 mM of sodium dodecyl sulfate (SDS) to the buffer solution in order to mimic the effect of the membrane [27].

RP-HPLC: The catalytic oxidation of DA promoted by Fe^III^-peptide complexes was studied at 37 °C in 10 mM Mops buffer pH 7.0 with 100 mM NaCl as the ionic strength buffer. The reaction was followed through DA quantification by isocratic RP-HPLC at 0, 1.5, 3, 6, and 24 h. Chromatographic separations were carried out by adapting the protocol reported by Sun et al. [28]. A solution made of 98% H_2_O, 0.1% HCOOH, and 2% CH_3_CN was used as the mobile phase, while the C18 Supelco Ascentis Express 100 × 4.6 mm column was used as the solid phase. In these conditions, DA retention time was 5.8 min. A 0–250 µM calibration curve was also prepared. For DA autoxidation, in a plastic tube covered with tinfoil, DA was added to a final volume of 10 mL of buffer (DA final concentration 200 µM). A 20 µL aliquot of this solution was withdrawn and injected into the HPLC system. The tube was covered with a holed sheet of tinfoil and left in a thermostatic bath at 37 °C. The procedure was repeated at the selected times. The effects of Fe and Fe^III^-peptide complexes were evaluated by adding 10 µM of metal ion and 20 µM of selected peptide, with iron as the last reagent. The procedure was also repeated with a lower DA:Fe ratio of 2:1 by adding 100 µM of the metal ion and 200 µM of the selected peptide.

HPLC-MS: The samples analyzed with RP-HPLC were also subjected to HPLC-MS analysis in order to identify peptide modifications. Samples at 0, 6, and 24 h were diluted with buffer to a final peptide concentration of 20 µM and analyzed in negative-ion mode.

#### 2.3.4. Kinetic Studies on DA Oxidation Promoted by Cu^II^-Peptide Complexes

The catalytic oxidation of DA (3 mM) promoted by Cu(II)-peptide complexes was studied at 37 °C in 25 mM Hepes buffer pH 7.4, following the absorbance of aminochrome at λ_max_ = 475 nm, according to the procedure reported previously [29]. The reaction rates were evaluated with 25 µM Cu^II^ and increasing amounts of Ac-αS_119–132_ (0–2 eq vs. copper(II)), with copper added as the last reagent.

#### 2.3.5. Identification of αS-Peptides Modification by HPLC-MS

Chemical modifications of Ac-αS_119–132_ promoted by Fe^III^-DA complexes were studied at 37 °C in 10 mM Mops buffer pH 7.0 with 100 mM NaCl as the ionic strength buffer. The reaction was followed every 90 min by HPLC-MS for 6 h, in negative-ion mode. The Ac-αS_119–132_ oxidation by buffer, Fe^III^ and DA were firstly considered. In a dark vial, 20 µM of Ac-αS_119–132_ were added to the buffer, loaded into the HPLC sample holder, and thermostated to 37 °C. Fractions of 100 µL were automatically injected into the HPLC systems every 90 min and analyzed. The effect of DA was taken into account by adding 200 µM of DA into the vial. Last, the effect of Fe^III^-DA complexes was evaluated by adding 10 µM of Fe^II^ as the last reagent. The whole protocol was repeated with a lower DA:Fe ratio of 2:1 by adding 20 µM of DA and 10 µM of Fe^II^. The effect of Ac-αS_119–132_ concentration was evaluated by preparing two samples: one with 40 µM peptide, 10 µM Fe^II^, and 200 µM DA, and another sample with a lower DA:Fe ratio of 2:1 with 20 µM of DA. The samples were incubated at 37 °C in a thermostated bath for 6 h. Later, the samples were diluted to a peptide final concentration of 20 µM and analyzed. The whole protocol was also repeated for Ac-αS^p^S_119–132_.

#### 2.3.6. Ac-αS_119–132_ Enzymatic Oxidation

The enzymatic oxidation of Ac-αS_119–132_ was carried out in 10 mM Mops buffer pH 7.0 with 100 mM NaCl as the ionic strength buffer, at 37 °C, using HRP. In a dark vial were added 20 µM of Ac-αS_119–132_, 0.2 µM of HRP, and 1.5 mM of hydrogen peroxide, as the last reagent, and the mixture was left at 37 °C for 2 h. Then, 100 µL of the sample were taken and analyzed through HPLC-MS in negative-ion mode.

## 3. Results and Discussion

### 3.1. Effect of αS C-Terminal Peptides on Fe^III^-DA Interaction

The effect of αS model peptides on the interaction between iron and DA was investigated in 10 mM Mops buffer pH 7.0 by varying both the DA:Fe ratio and the peptide concentration. The selected pH was slightly acidic compared to the physiological pH due to the tissue acidosis found in PD brains [7,10]. The combined use of iron and DA gives rise to some problems: (i) the well-known propensity of DA to form insoluble DA-quinone oligomers upon oxidation; (ii) the strong competition of Fe^III^ hydrolysis at pH above 4 in aerated solutions [30]; and (iii) the formation of the bis-catecholato-Fe^III^ complex [Fe^III^(DA)_2_]^–^, which has a strong LMCT band in the visible region (λ_max_ = 580 nm, ε_580_ = 3300 M^−1^ cm^−1^) [9,31] thus preventing observation of the UV-Vis band of aminochrome (λ_max_ = 475 nm), the observable product of DA oxidation [32]. Therefore, the experiments with Fe–DA-αS model peptides were monitored through the formation and decay of the [Fe^III^(DA)_2_]^–^ species at 580 nm.

**Figure 2:**
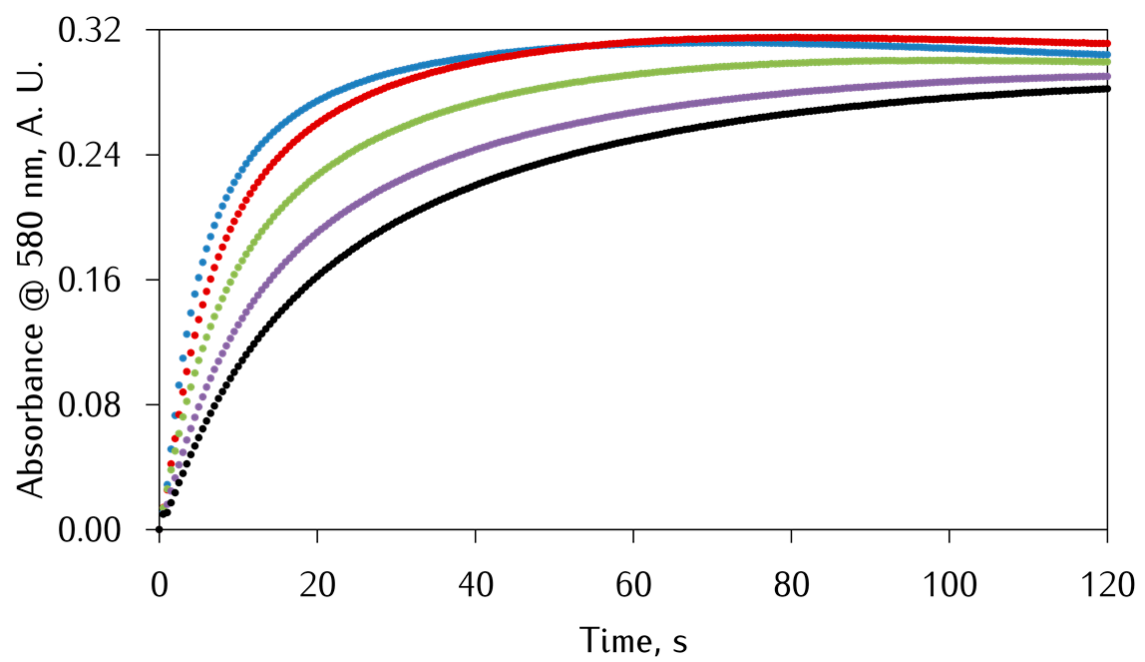
Kinetic traces of DA-Fe^II^ (2:1) interaction as a function of Ac-αS_119–132_ concentration (λ_max_ = 580 nm) in 10 mM Mops buffer pH 7.0, 37 °C, Fe^II^ 0.1 mM, DA 0.2 mM (blue), and increasing amounts of Ac-αS_119–132_ (0.5 equiv.: red, 1 equiv.: green, 1.5 equiv.: purple, 2 equiv.: black).

**Scheme 3:**
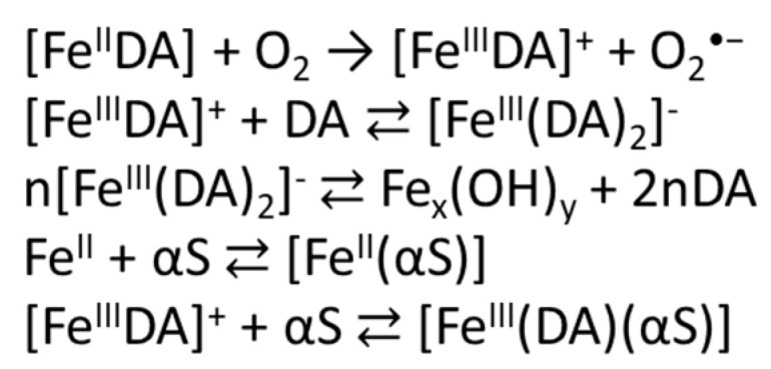
Proposed mechanism for [Fe^III^(DA)_2_]^–^ complex formation and degradation [9]. αS peptides compete with DA towards iron binding with either Fe^II^ or [Fe^III^DA]^+^.

**Figure 3:**
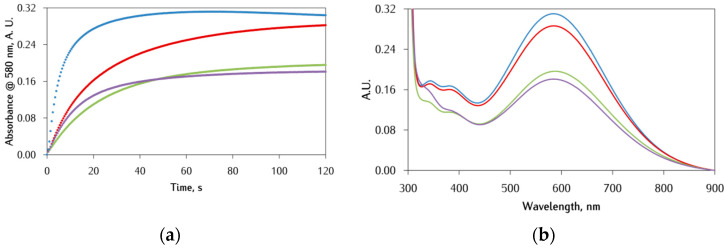
(**a**) Kinetic traces (λ_max_ = 580 nm) and (**b**) UV-Vis spectra of DA-Fe^II^ (2:1) interaction as a function of peptide concentration in 10 mM Mops buffer pH 7.0, 37 °C, Fe^II^ 0.1 mM, DA 0.2 mM (blue), and 2 equiv. of peptide (Ac-αS_119–132_: red, Ac-αS^p^S_119–132_: green, Ac-αS^p^Y^p^S_119–132_: purple).

First, the effect of Ac-αS_119–132_ on the formation and decay of [Fe^III^(DA)_2_]^–^ was evaluated, at a DA:Fe ratio of 2:1, and the kinetic traces of absorbance against time are reported in Figure 2. The obtained kinetic traces display a biphasic behavior due to the complex mechanism described below. As reported by Sun et al. [9], upon mixing Fe^II^ and DA, the initial Fe^II^-DA complex is readily oxidized by dioxygen resulting in superoxide radical anion and Fe^III^-DA; this is then converted in the [Fe^III^(DA)_2_]^–^ complex which is responsible for the strong absorption in the visible region (Scheme 3). The growth rate of the 580 nm band depends on the DA:Fe ratio but, at the lowest ratio (i.e., 2:1), Fe^III^ hydrolysis is competitive [16] and, thus, the [Fe^III^(DA)_2_]^–^ complex decomposes causing the decrease of the complex concentration and, consequently, of the kinetic traces slope. When the experiment was carried out in the presence of Ac-αS_119–132_, a decrease in both the final concentration and formation rate of the [Fe^III^(DA)_2_]^–^ complex was observed. It is worth noting that these effects are greater as the peptide concentration increases. This effect could be explained considering the establishment of a competition between the peptide and the DA towards iron(II) or iron(III) binding, hence reducing the amount of the [Fe^III^(DA)_2_]^–^ complex. Nevertheless, peptide binding seems to also affect the second phase of the reaction, decreasing the rate of iron(III) hydrolysis. Further information can be extracted from the UV-Vis spectra (Figure 3) recorded during the kinetic study, in which a red-shift of ca. 15 nm of the LMCT band suggests that the Ac-αS_119–132_ reaction with the [Fe^III^(DA)_2_]^–^ complex is likely to occur, forming a ternary complex [Fe(DA)(Ac-αS_119–132_)] (Figure 1) showing the characteristic band of the Fe^III^-monocatecholato complex [Fe^III^DA]^+^. This last assumption is in good agreement with the results reported by Jiang et al., which claim that the formation of the ternary complex [Fe^III^(DA)(ATP)] is followed by a marked red shift of the LMCT band [33].

The phosphorylated peptides Ac-αS^p^S_119–132_ and Ac-αS^p^Y^p^S_119–132_ have a stronger impact on the formation and degradation rate of the [Fe^III^(DA)_2_]^–^ complex as, in both cases, the rates decrease more significantly than with the non-phosphorylated analogue (Figure 3). Furthermore, the shift towards lower energies of the LMCT band is slightly larger compared to the one caused by the non-phosphorylated analogue (Figure 3), also suggesting the formation of the ternary complex. This finding is in line with the gain in the stability constant of the peptides for Fe^III^ binding, offered by the phosphorylation of S129 and Y125 [13,14,20]. Compared to the kinetic trace of Ac-αS^p^S_119–132_, it is worth noting that the kinetic trace of Ac-αS^p^Y^p^S_119–132_ appears to be different: the initial rate of the [Fe^III^(DA)_2_]^–^ complex formation is higher, but the final amount of complex is lower. We hypothesize that this phenomenon is caused by an initial competition between the two phosphorylated sites due to difficult conformational changes needed for Fe^III^ coordination coupled with the negative repulsion offered by two phosphate groups, with the pY125 being the strongest ligand [14], thus resulting in a lower final concentration of the [Fe^III^(DA)_2_]^–^ complex. 

**Figure 4:**
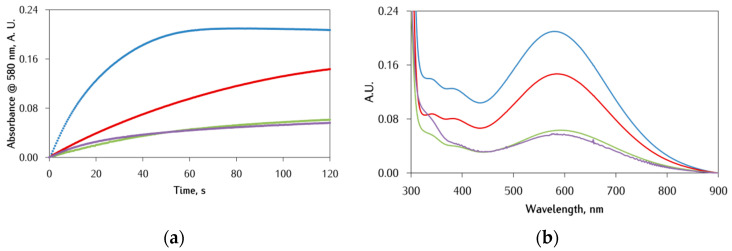
(**a**) Kinetic traces (λ_max_ = 580 nm) and (**b**) UV-Vis spectra of DA-Fe^II^ (2:1) interaction as a function of peptide concentration in 10 mM Mops buffer and 20 mM SDS, pH 7.0, 37 °C, Fe^II^ 0.1 mM, DA 0.2 mM (blue), and 2 equiv. of peptide (Ac-αS_119–132_: red, Ac-αS^p^S_119–132_: green, Ac-αS^p^Y^p^S_119–132_: purple).

Since the interaction of αS peptides with lipid membrane modulates their conformation, analysis of the interaction between iron and αS is incomplete if it remains confined in the aqueous *milieu*. For this reason, the anionic surfactant SDS is used in this investigation because the negative surface charge and high affinity to hydrophobic domains of the micelles formed above the critical concentration (≈8 mM in water at 25 °C) [34,35] represent a useful and simple model of biological membranes. The addition of SDS (Figure 4) to the buffer enhances the effect of αS peptides over [Fe^III^(DA)_2_]^–^ formation and consumption, making the slope of the first phase of the kinetics much less steep. A possible explanation could be that, at the investigated pH (i.e., 7.0), the DA aromatic hydroxyl group is protonated and, thus, the aromatic ring can be inserted into the SDS micelles [27] causing a decrease in the effective DA concentration and making DA less competitive towards Fe^III^ binding compared to the αS peptides.

Similar conclusions were drawn after performing the experiments at the 20:1 DA:Fe molar ratio (Figure S1). The main difference highlighted upon increasing the DA concentration is the diminution of the peptide effect. This aspect was expected since the large DA excess and its high affinity for Fe^III^ binding hampers the interaction of αS-peptides with the [Fe^III^(DA)_2_]^–^ complex.

### 3.2. Kinetic Studies on the Oxidation of Catechol Substrates Promoted by Fe^III^-Peptide Complexes

The catalytic oxidation of DA promoted by Fe-αS peptides was studied in Mops buffer at pH 7.0, at the two different DA:Fe ratios of 2:1 and 20:1, adding 2 equiv. of Ac-αS_119–132_, Ac-αS^p^S_119–132_, or Ac-αS^p^Y^p^S_119–132_. In order to overcome the difficulties related to monitoring Fe^III^-DA chemistry, different techniques were applied—i.e., RP-HPLC, UV-Vis spectroscopy, and HPLC-MS/MS. Due to the intense [Fe^III^(DA)_2_]^–^ LMCT band, it is not possible to follow DA oxidation through the development of the absorption band of aminochrome at 475 nm, which accumulates in the reaction initial stages. Furthermore, the DA oxidation products can polymerize and form insoluble aggregates with a general increase of baseline absorption over the whole wavelength range of the spectrum due to light scattering. For these reasons, DA oxidation was monitored following a decrease of the absorbance of DA (λ_max_ = 280 nm) over time, removing background contribution by a three-point-dropline correction.

**Figure 5:**
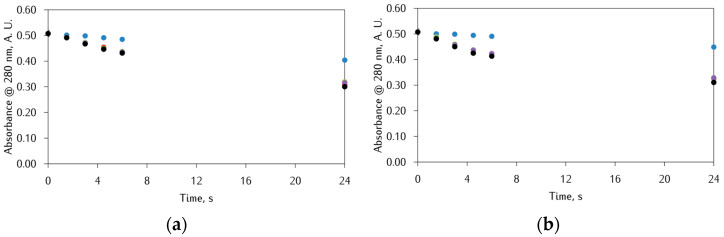
(**a**) Kinetic traces of 200 µM DA in Mops buffer pH 7.0 and 37 °C (blue) in the presence of 10 µM of Fe^II^ (red) and 2 equiv. of peptide (Ac-αS_119–132_: green, Ac-αS^p^S_119–13_: purple, Ac-αS^p^Y^p^S_119–132_: black). (**b**) Kinetic traces of 200 µM DA in Mops buffer pH 7.0, SDS 20 mM, 37 °C (blue) in the presence of 10 µM of Fe^II^ (red) and 2 equiv. of peptide (Ac-αS_119–132_: green, Ac-αS^p^S_119–13_: black).

**Figure 6:**
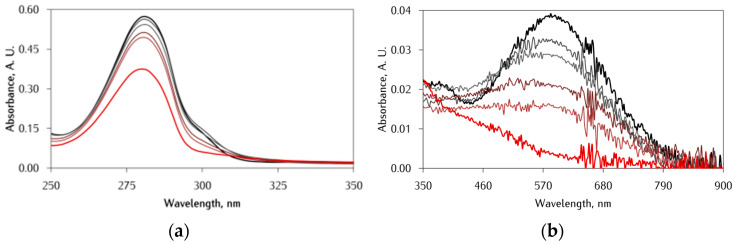

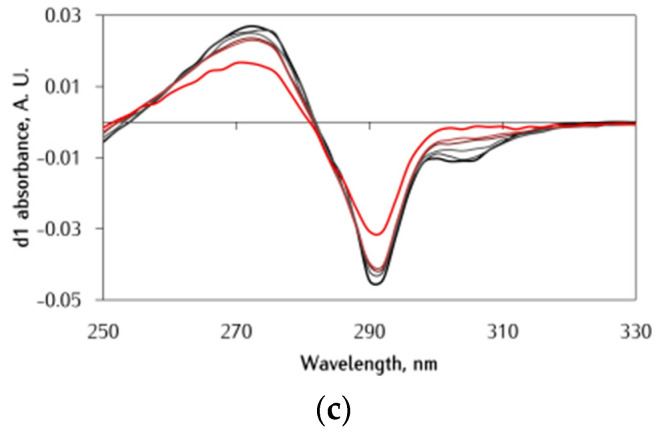
UV-Vis spectra of DA oxidation kinetics by Fe^III^-Ac-αS_119–132_ complex (DA:Fe 20:1) in 10 mM Mops buffer pH 7.0 and 37 °C; progression of time is rendered as color hue transition from black (0 h) towards red (24 h). Spectra were recorded at 0, 1.5, 3.0, 4.5, 6, and 24 h. (**a**) 250–350 nm region magnification of UV-Vis spectra. (**b**) 350–900 nm region magnification of UV-Vis spectra. (**c**) UV-Vis spectra first derivative.

Starting from the experiments at the higher DA:Fe molar ratio of 20:1, the kinetic traces of absorbance against time are reported in Figure 5, which refers to DA oxidation by Fe^III^-αS-peptides. The results clearly show that the catalytic oxidation of DA by iron is not significantly promoted by the addition of either Ac-αS_119–132_, Ac-αS^p^S_119–132,_ or Ac-αS^p^Y^p^S_119–132_, since the kinetic traces obtained with the peptides are almost identical to the control experiment without peptides, in line with what is expected. In fact, as seen for the Fe–DA coordination experiments, a large excess of DA makes the interaction of αS-peptides with the metal center difficult. Furthermore, comparing the UV-Vis spectra obtained with and without αS-peptides (Figure 6), the following similarities are observed: (i) the DA absorption band (λ_max_ = 280 nm) and the [Fe^III^(DA)_2_]^–^ band (λ_max_ = 580 nm) show a similar decrease; (ii) the [Fe^III^(DA)_2_]^–^ complex band shows the same shape, and at 6 h the available Fe^III^ is still bound to the DA; (iii) the DA band has a shoulder at around 305 nm, better highlighted in the first derivative spectra (panel C), which is due to the DA fraction bound to Fe^III^ [36]; and (iv) the band intensity decrease is similarly followed by a progressive background increase in both cases, due to the melanic pigment particle formation [37].

Further, DA oxidation was also followed by RP-HPLC (Figure S2). As in the UV-Vis experiments, the chromatographic data show no difference in DA oxidation rates between the control experiment and the experiments in the presence of αS.

The addition of SDS (Figure 5–panel B) to the buffer caused a slight increase in global DA oxidation rate in all samples. We hypothesize that this effect is driven by the possible interaction between SDS and DA oligomers. In the UV-Vis spectra in the presence of SDS (Figure S3), an increase of the background absorption in the first 3 h is observed, followed by a decrease from 3 to 6 h due to the formation of melanic aggregates. These aggregates may stick to the SDS micelles [27], thus accumulating in the solution before precipitating as melanin particles. Consequently, the melanic pigments do not bind iron which can, in turn, catalyze DA oxidation.

**Figure 7:**
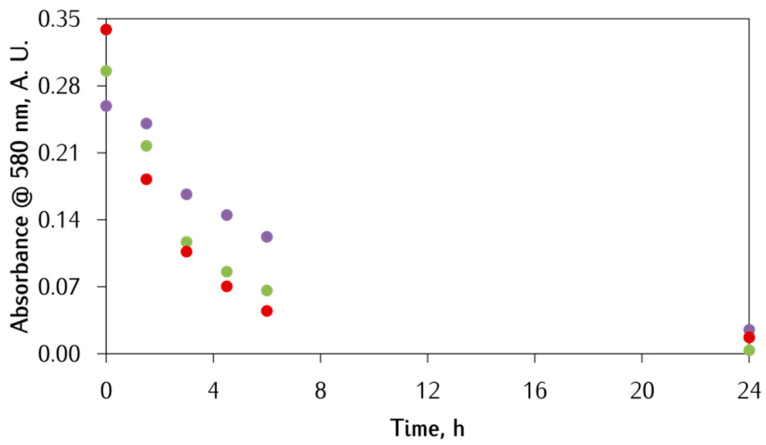
Kinetic traces of DA (200 µM) oxidation in Mops buffer pH 7.0 and 37 °C in the presence of 100 µM of Fe^II^ (red) and 200 µM (2 equiv.) of peptide (Ac-αS_119–132_: green, Ac-αS^p^S_119–132_: purple).

Considering the experiments at the lower DA:Fe molar ratio of 2:1, a different situation is observed. Herein, DA oxidation cannot be reliably monitored following the absorbance of DA (λ_max_ = 280 nm) over time, because this band is altered by coordination to Fe^III^ and overlapped with the peptide tyrosine absorption band (Figure S4). Therefore, the LMCT band of the [Fe^III^(DA)_2_]^–^ complex at 580 nm was monitored instead. In Figure 7, the kinetic traces of absorbance against time are reported for DA oxidation by Fe^III^-Ac-αS_119–132_. The starting points of the kinetic traces are different among the samples due to the impact of the peptides on the [Fe^III^(DA)_2_]^–^ complex, already discussed in the previous section. Nevertheless, the results clearly indicate that addition of Ac-αS_119–132_ and particularly Ac-αS^p^S_119–132_ causes a decrease in the DA oxidation rate, as suggested by the lower slope of the traces, probably due to the [Fe(DA)(Ac-αS_119–132_)] ternary complex formation (Figure 1) which stabilizes the Fe^III^ oxidation state, or by hampering DA binding and thus redox cycling.

**Figure 8:**
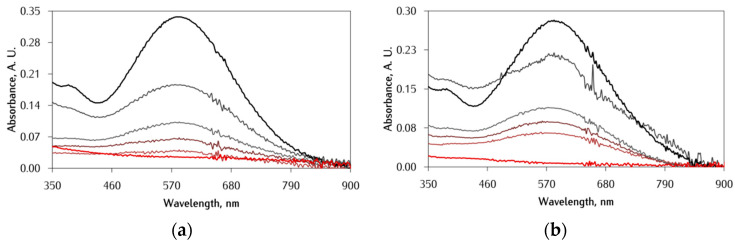
(**a**) UV-Vis spectra of DA oxidation at various times by Fe^II^ (DA:Fe 2:1) in 10 mM Mops buffer pH 7.0 and 37 °C; progression of time is rendered as color hue transition from black (0 h) towards red (24 h). Spectra were recorded at 0, 1.5, 3.0, 4.5, 6, and 24 h. (**b**) UV-Vis spectra of DA oxidation progress by Fe^III^-Ac-αS_119–132_ complex (DA:Fe:peptide 2:1:1) in 10 mM Mops buffer pH 7.0 and 37 °C, progression of time is rendered as color hue transition from black (0 h) towards red (24 h). Spectra were recorded at 0, 1.5, 3.0, 4.5, 6, and 24 h.

In the UV-Vis spectra with and without Ac-αS_119–132_, reported in Figure 8, the main difference appears to be the higher absorption in the 350–500 nm portion of the 1.5 h spectra. Even if we do not know the nature of this spectral feature, it may be related to peptide-promoted Fe^III^ aggregation or to a form of melanic intermediate. Both are in line with the reduced oxidation rate of DA.

Also in this case, DA oxidation was followed by RP-HPLC (Figure S5). In agreement with UV-Vis spectral changes, the chromatographic data show a reduction in DA oxidation rate upon the addition of αS-peptides, but no significant difference was found between Ac-αS_119–132_ and Ac-αS^p^S_119–132_.

### 3.3. Identification of αS-Peptide Modifications by HPLC-MS

It is well known that upon metal-catalyzed oxidation of catechols such as DA, ROS species are generated [7,38,39]. Such harmful species include O_2_^•−^, H_2_O_2_, and OH^•^, which ultimately lead to protein damage. Based on the results obtained so far, one important target of this damage are the αS-peptides themselves due to their ability to influence both the [Fe^III^(DA)_2_]^–^ complex formation and degradation. Furthermore, studies on the peptide modification could also shed light on the type of interaction and on the mechanism of reaction. The modifications undergone by Ac-αS_119–132_ and Ac-αS^p^S_119–132_ concomitantly with DA oxidation were identified by performing a series of HPLC-MS/MS analyses. The experiments were run in 10 mM Mops buffer pH 7.0, by varying both the DA:Fe ratio and the peptide concentration. Typical oxidative modifications usually consist of an O-atom insertion (+16 Da) in oxidation-sensitive residues such as tyrosine and methionine, which are converted into 3-hydroxytyrosine and methionine sulfoxide, respectively [40]. Unfortunately, the mass spectra recorded in negative-ion mode fail to be fed into algorithms like turboSEQUEST and, hence, the MS/MS spectra had to be analyzed by inspection.

#### 3.3.1. Ac-αS_119–132_ Peptide

**Figure 9:**
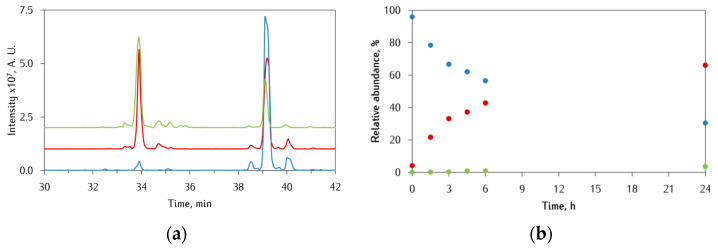
(**a**) HPLC-MS elution profiles of Ac-αS_119–132_ (20 µM) in Mops buffer pH 7.0 at 37 °C, upon reaction with Fe^II^ (10 µM) and DA (20 µM) at the beginning (blue), and after 6 h (red) and 24 h of incubation (green). (**b**) Per cent modification detected by the HPLC-MS analysis: Ac-αS_119–132_ (t_r_ 39 min, blue), Ac-αS_119–132_ +16 (t_r_ 33.8 min, red), Ac-αS_119–132_ +32 (t_r_ 35.2 min, green).

When the Ac-αS_119–132_ peptide was used at the lower DA:Fe ratio of 2:1, the elution profiles of Ac-αS_119–132_ together with the modifications detected by the HPLC-MS/MS analysis were obtained as shown in Figure 9. After 6 h of incubation, the elution profile is dominated by two main species: (i) the unmodified Ac-αS_119–132_ peptide (t_r_ 39 min, 56%) and (ii) the single oxidized derivative (t_r_ 33.8 min, 43%). After 24 h, the overall amount of oxidized peptide increased (66%) and a new species was detected at t_r_ 35.2 min (3.5%), which appears to be the double oxidized peptide. Together with the above species, other minor modifications were detected, mainly related to peptide truncation. However, due to the low intensities of such signals, it was not possible to assess the exact nature of the corresponding species. Unfortunately, none of the peptide-DA and DA-quinone oligomeric species could be detected, probably due to the lack of strong nucleophilic residues in the peptide. Considering the peptide primary sequence, the most likely sites of oxidation are Y125 and M127. However, from the analysis of the ion fragmentation series found in the MS/MS spectra, it was not possible to assess the exact oxidation site. To overcome this problem, the peptide was selectively oxidized by hydrogen peroxide and horseradish peroxidase, which can differentiate the two amino acids by promoting the methionine sulfoxidation and tyrosine dimerization. The elution profile obtained showed an intense peak at t_r_ 33.6 min with the same MS/MS fragmentation patterns of the mono oxidized Ac-αS_119–132_; hence. we can easily conclude that the oxidation occurs at M127 since HRP does not catalyze tyrosine hydroxylation but its dimerization. Based on this finding, the second oxidation of the peptide could occur either at the oxidized M127 (to form sulfone) or at the Y125. Furthermore, control experiments were carried out by incubating the peptide alone and in the presence of only DA or only Fe^II^. The data obtained showed no appreciable oxidation of M127 when the peptide was incubated alone in the buffer or with only DA. On the other hand, in the case of only Fe^II^, the single oxidized peptide was only detected after 90 min, albeit being less than 10%. 

When the reaction was carried out at the higher DA:Fe molar ratio of 20:1, the elution profiles of Ac-αS_119–132_ and the peptide modifications detected by HPLC-MS shown in Figure S6 were obtained. Also in this case, as seen above for the DA oxidation, the increase in the DA:Fe ratio is followed by a decrease of the observed modification, being 15% at 6 h and 28% at 24 h.

**Scheme 4:**
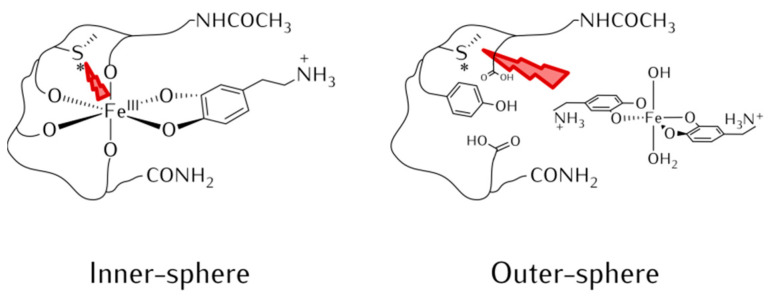
The possible oxidation mechanisms of Ac-αS_119–132_ upon Fe^III^-DA promoted oxidation: left, *inner-sphere*, where the bound peptide is oxidized; right: *outer-sphere*, where the unbound peptide is oxidized.

To further investigate both the type of Fe-peptide interaction and the peptide modification mechanism, the experiments were replicated by doubling the peptide concentration (40 µM) for the studied case. Considering the possible scenarios, two representative cases were considered: (i) the peptide oxidation occurs through an *outer-sphere* (OS) mechanism, where the peptide is not directly bound to the iron site, or (ii) the oxidation occurs through an *inner-sphere* (IS) mechanism and, therefore, it undergoes oxidation when bound to iron (Scheme 4). As a rule of thumb, in the former case, by doubling the peptide concentration, we expect to observe the same percentage of modification, while, in the latter case, a lower (a halving in the case of strong binding) of the percentage is expected. However, it is worth noting that the two mechanisms could be operative at the same time, with IS generally more efficient. The data obtained (Table 1) suggest an overlap between the two mechanisms depending on the DA:Fe ratio. In fact, at the lower DA:Fe ratio, upon doubling the peptide concentration, a 25% decrease of oxidized peptide was observed, thus suggesting an initial IS mechanism with the excess peptide being oxidized through an OS mechanism, which accounts for the observed decrease of the percentage. At the higher DA:Fe ratio, an initial OS mechanism is likely to occur as the percentage of oxidized peptide is lower, even though we cannot exclude that the mechanism could be IS-type but with a low fraction of the peptide coordinated. By doubling the peptide concentration, a doubling in the modification percentage is observed, suggesting that the higher Ac-αS_119–132_ concentration leads to an increase of the coordinated fraction which will be oxidized faster through the IS mechanism.

We can, therefore, conclude that αS-peptide oxidation is likely to occur through an IS-type mechanism, in which the coordinated peptide is oxidized, while the OS mechanism could contribute only to a modest extent. These findings are in line with what was found in the previous sections, where a strong interaction between Ac-αS_119–132_ and [Fe^III^(DA)_2_]^–^ can only occur at a low DA:Fe ratio.

#### 3.3.2. Ac-αS^p^S_119–132_ Peptide

**Figure 10:**
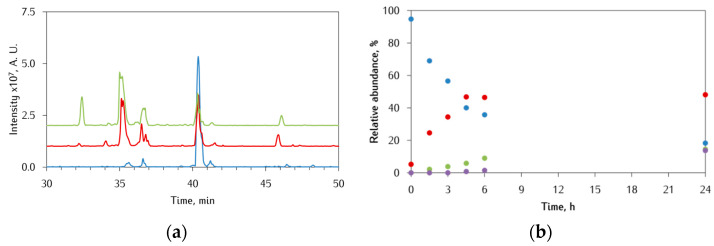
(**a**) HPLC-MS elution profiles of Ac-αS^p^S_119–132_ (20 µM) in Mops buffer pH 7.0 at 37 °C, in the presence of Fe^II^ (10 µM) and DA (20 µM) at the beginning (blue), and after 6 h (red) and 24 h of incubation (green). (**b**) Per cent modification detected by HPLC-MS analysis: Ac-αS^p^S_119–132_ (t_r_ 40 min, blue), Ac-αS^p^S_119–132_ +16 (t_r_ 34.6 min, red), Ac-αS^p^S_119–132_ +32 (t_r_ 36.4 min, green), Ac-^119^DPDNEAYEM*^127^ fragment (t_r_ 32 min, purple).

Considering the oxidation undergone by the phosphorylated peptide Ac-αS^p^S_119–132_, Figure 10 shows the elution profiles together with the modifications detected by the HPLC-MS/MS analysis, at the lower DA:Fe ratio of 2:1. As discussed in the previous sections, phosphorylation of S129 has a strong impact on the ability of Ac-αS^p^S_119–132_ to compete with DA for the interaction with the [Fe^III^(DA)_2_]^–^ complex. In fact, the unmodified peptide (t_r_ 40 min) found in solution is 35% at 6 h of incubation, with a further decrease to less than 20% after 24 h. Contrary to the previous case, the oxidative modifications appear to be dominant species, especially at 24 h. Among them, it is worth noting the presence of the double oxidized peptide (t_r_ 36.4 min, 15%) and several truncated species. The exact nature of such species is currently undefined, but we hypothesize that the species eluted at t_r_ 32 min (14%) corresponds to the truncation of the mono oxidized peptide, with sequence Ac-^119^DPDNEAYEM*^127^, where M127 is oxidized to sulfoxide. This latter peptide fragment suggests that the presence of the phosphate group on S129 causes a shift of the metal coordinating region towards the peptide C-terminus.

At the higher DA:Fe ratio of 20:1, a decrease in the oxidation rate of Ac-αS^p^S_119–132_ was observed (Figure S7), even though it was less pronounced compared with that of Ac-αS_119–132_, again in agreement with the higher affinity towards Fe^III^ contributed by the phosphoserine pS129. Interestingly, after 24 h of incubation, two new peaks appeared at t_r_ 38.6 and 31.8 min, which share the same MS/MS pattern of the Ac-αS_119–132_ peptide and the corresponding mono oxidized product, respectively; hence, dephosphorylation of pS129 is likely to occur.

Upon doubling the concentration of Ac-αS^p^S_119–132_ peptide to 40 µM, the data obtained (Table 2) suggest that the oxidation mechanism still depends on the DA:Fe ratio but appear to be less ambiguous. In fact, at the lower ratio of 2:1, after doubling the peptide concentration, a halving of the per cent of oxidized peptide was observed, indicating an IS mechanism. At the higher ratio of 20:1, an OS mechanism occurs instead since the percentage of oxidized peptide remained almost unchanged with only a small decrease probably related to the competitive peptide truncation. Similar conclusions can be drawn for Ac-αS^p^S_119–132_ when compared to Ac-αS_119–132_. Again, serine phosphorylation has a strong impact on the peptide effect over Fe–DA chemistry since, at both DA:Fe ratios, a greater percentage of peptide modification and truncation was found, in line with a stronger αS-peptide-Fe interaction.

### 3.4. Comparison with Copper

Besides iron, it is well known that copper is able to promote DA oxidation in the presence of neuronal peptides [41]. It is, thus, useful to compare the behavior of the two metal ions bound to the same αS peptide. Following our previous studies on the ability of Cu^II^-Aβ16 complexes to promote catechol oxidation [29], we aimed to investigate whether αS, and in particular the Ac-αS_119–132_ peptide, can catalyze this reaction. In fact, the protein region contains a binding site for divalent metal ions in the ^119^DPDNEA^124^ motif [12]. 

**Figure 11:**
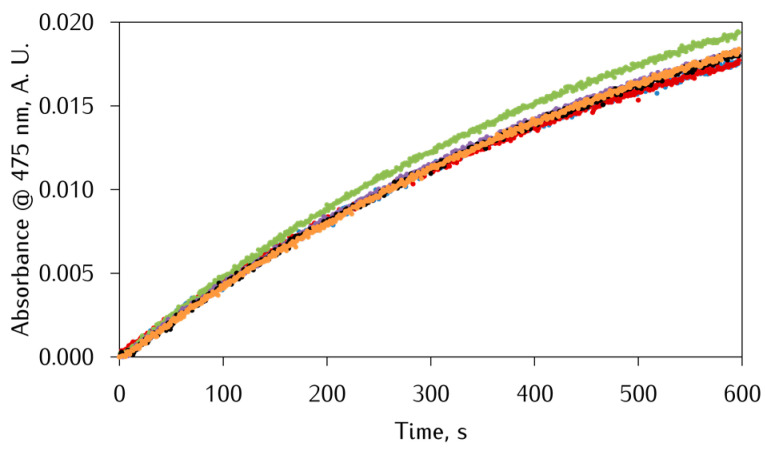
Kinetic traces of 3 mM DA in Hepes buffer pH 7.4 and 37 °C (blue) in the presence of 25 μM of Cu(II) (red) and increasing amounts of Ac-αS_119–132_ (0.5 eq: green, 1 eq: purple, 1.5 eq: black, 2 eq: orange). The DA autoxidation (without copper or peptide) overlaps with the lower, purple, trace.

The catalytic oxidation of DA by Cu(II)-Ac-αS_119–132_ complex was studied in Hepes buffer at pH 7.4 adding increasing amounts of Ac-αS_119–132_. The kinetic traces of absorbance against time are reported in Figure 11, where the readings at 475 nm refer to the formation of aminochrome [29]. As can be observed from Figure 11, catalytic oxidation of DA by Cu^II^ is not promoted by the addition of Ac-αS_119–132_, since kinetic traces obtained with the peptide are almost identical. These findings are even clearer by plotting the slope of the kinetic traces against equivalents of peptide added, as reported in Figure S8. Again, since the slope is quite constant over the peptide addition, we can conclude that Ac-αS_119–132_ is neither able to catalyze nor suppress DA oxidation.

## 4. Conclusions

The interaction between C-terminal fragments of αS with biologically relevant metal ions and DA was investigated. The data suggest that Ac-αS_119–132_ and particularly Ac-αS^p^S_119–132_ and Ac-αS^p^Y^p^S_119–132_ are able to interact with the [Fe^III^(DA)_2_]^–^ complex, likely forming a ternary complex—[Fe(DA)(αS)]. The interplay between the three species is dominated by two factors: (i) the DA:Fe molar ratio and (ii) the phosphorylation of the peptide. At high DA:Fe molar ratios, DA coordination is too strong and hampers the peptide coordination, while, at lower DA:Fe molar ratios, the peptide is able to compete with one of the two coordinated DA molecules. This kind of interaction is also confirmed by the analysis of the post-translational modifications (PTMs) of the peptide, where an inner-sphere mechanism was observed only at the DA:Fe ratio of 2:1. Nevertheless, peptide phosphorylation enhances affinity for Fe^III^, causing a decrease in DA oxidation rate and increase in peptide oxidation. Furthermore, phosphorylation at S129 causes the shift of the coordination sphere towards the C-terminus of the peptide, as a consistent fraction of fragmented peptide was observed. 

Studies conducted in a membrane-like environment showed an enhanced peptide effect over both the DA oxidation and the [Fe^III^(DA)_2_]^–^ complex formation and degradation by lowering the effective DA concentration and thus shifting the DA:Fe molar ratio towards lower values. 

In the case of copper(II), we demonstrated that the Ac-αS_119–132_ peptide does not influence the DA oxidation promoted by the free ion. This behavior contrasts with what was observed with other neuronal peptides like Aβ and prion fragments [41], and is due to the lack of free *N*-terminal amine and histidine in the *C*-terminal αS, which are the residues that typically offer a strong Cu^II^ coordination sphere and stabilize the Cu^I^ redox state necessary to cycle during the catalytic event.

Future perspectives will address the full characterization of the bis-phosphorylated αS fragment at both Y125 and S129. Despite that, the preliminary data indicate that the second phosphorylation (i.e., on Y125) does not enhance interaction with [Fe^III^(DA)_2_]^–^, if compared with the Ac-αS^p^S_119–132_ peptide, and rather a competition between the two residues occurs. A further aspect to be taken into consideration will be the use of more sophisticated membrane models than the micelles formed by SDS; for example, with large or small unilamellar vesicles (LUV and SUV, respectively). Since the membrane alters the effective DA concentration, other DA:Fe molar ratios than those used in the present work need to be investigated.

## Figures and Tables

**Table 1:** Effect of Ac-αS_119–132_ concentration on the extent of peptide oxidation.

DA:Fe:Peptide (Equiv.)	Incubation Time (h)	Ac-αS_119–132_ +16 (%)
2:1:2	6	43
2:1:4	6	33
20:1:2	6	15
20:1:4	6	28

**Table 2:** Effect of Ac-αS^p^S_119–132_ concentration over oxidation mechanism.

DA:Fe:Peptide (Mol Ratio)	Incubation Time (h)	Ac-αS^p^S_119–132_ +16 (%)
2:1:2	6	56
2:1:4	6	29
20:1:2	6	54
20:1:4	6	41

## Data Availability

Data supporting the reported results can be obtained from the corresponding author.

## Supplementary Materials

Additional kinetic traces (Figures S1, S2, S4, S5 and S8), additional UV-vis spectra (Figure S3), additional HPLC-MS elution profiles, and percentage modifications (Figures S6 and S7) tables with kinetic constants (Table S1) are available online and can be downloaded at: https://www.mdpi.com/article/10.3390/antiox12040791/s1.
