# Supplementary material for: Interaction and Redox Chemistry between Iron, Dopamine, and Alpha-Synuclein C-Terminal Peptides
Source: Antioxidants (Basel). 2023 Mar 24;12(4):791. doi: 10.3390/antiox12040791 (PMC10135331; doi:10.3390/antiox12040791)
Supplement: Supplementary file 1 [file antioxidants-12-00791-s001.zip › antioxidants-2276188-supplementary.pdf]

Supplementary materials: Interaction and Redox Chemistry Between Iron, Dopamine and Alpha-Synuclein C-Terminal Peptides

Fabio Schifano [1,2], Simone Dell'Acqua [1], Stefania Nicolis [1], Luigi Casella [1,*] and Enrico Monzani [1,*]

[1] Department of Chemistry, University of Pavia, Via Taramelli 12, 27100 Pavia, Italy; fabio.schifano01@universitadipavia.it (F.S.); simone.dellacqua@unipv.it (S.D.); stefania.nicolis@unipv.it (S.N.); enrico.monzani@unipv.it (E.M.); luigi.casella@unipv.it (L.C.)
[2] IUSS University School for Advanced Studies of Pavia, Palazzo del Broletto, Piazza della Vittoria 15, 27100 Pavia, Italy
* Correspondence: enrico.monzani@unipv.it (E.M.); luigi.casella@unipv.it (L.C.)

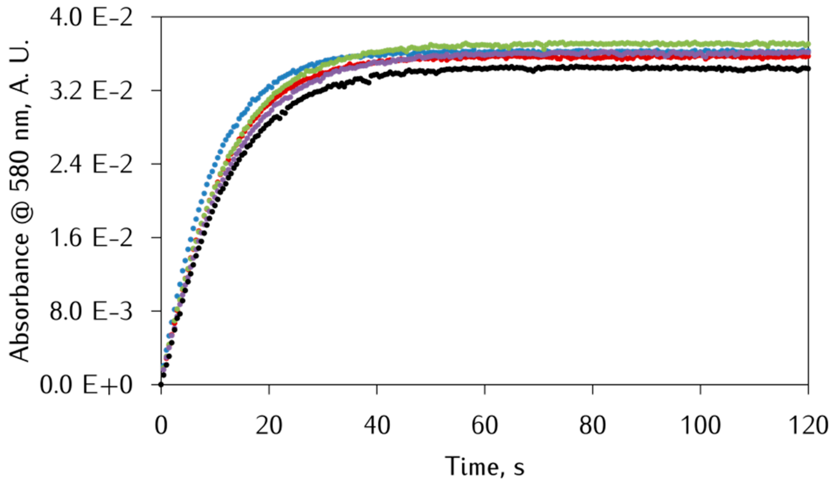

Figure S1. Kinetic traces of DA-Fe^{II} (20:1) interaction as a function of Ac-αS^{p}S$_{119-132}$ concentration (λ_{max} = 580 nm) in 10 mM Mops buffer pH 7.0 and 37 °C, iron(II) 10 μM, DA 0.2 mM (blue) and increasing amount of Ac-αS^{p}S$_{119-132}$ (0.5 equiv.: red, 1 equiv.: green, 1.5 equiv.: purple, 2 equiv.: black).

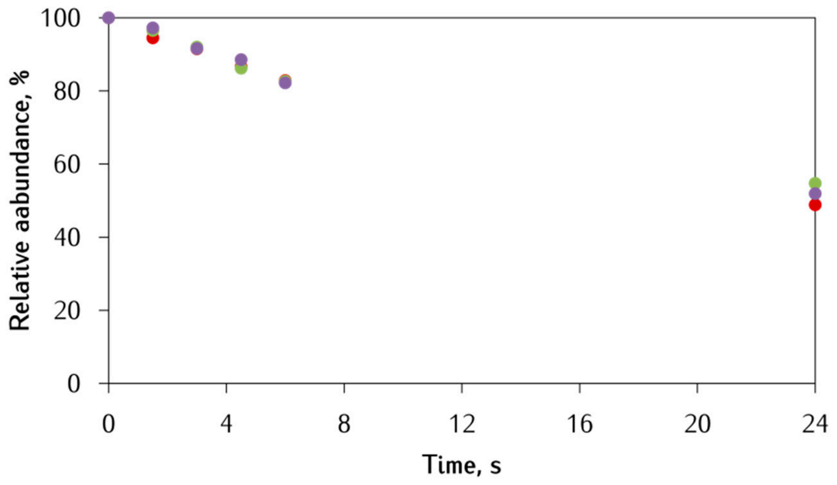

Figure S2. RP-HPLC kinetic traces of 200 μM DA in Mops buffer pH 7.0 and 37 °C (blue) in presence of 10 μM of iron(II) (red) and 2 equiv. of peptide (Ac-αS$_{119-132}$: green, Ac-αS^{p}S$_{119-132}$: purple, Ac-αS^{p}Y^{p}S$_{119-132}$: cyan).

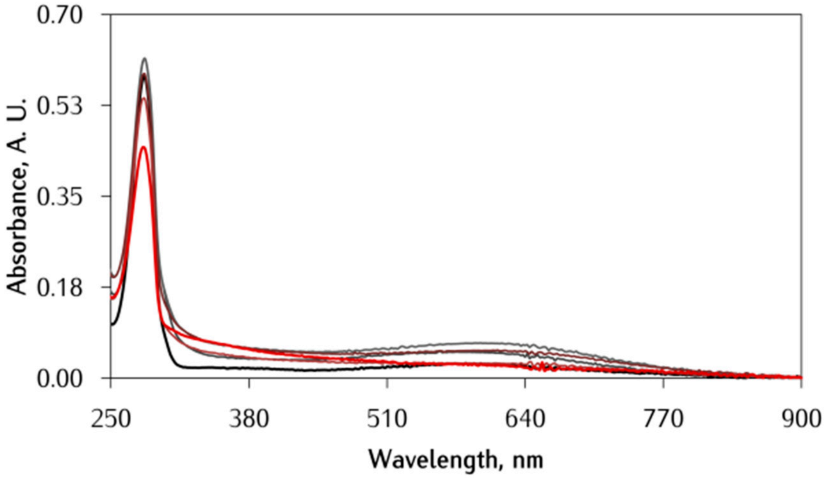

Figure S3. UV-Vis spectra of DA oxidation kinetics by Fe^{III}-Ac-αS119-132 complex (DA:Fe 20:1) in 10 mM Mops buffer pH 7.0 and SDS 20 mM, 37 °C; progression of time is rendered as color hue transition from black (0 h) towards red (24 h). Spectra were recorded at 0, 1.5, 3.0, 4.5, 6 and 24 h.

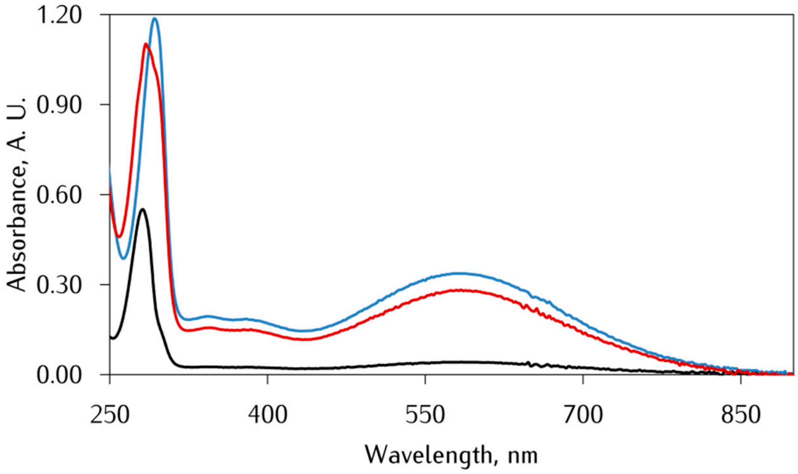

Figure S4. UV-Vis spectra of DA (200 μM) oxidation at 0 h in 10 mM Mops buffer pH 7.0 and 37 °C, in the presence of 10 μM of Fe^{II} (black), 100 μM of Fe^{II} (blue) or 100 μM Fe^{II} and 200 μM Ac-αS119-132 peptide (red).

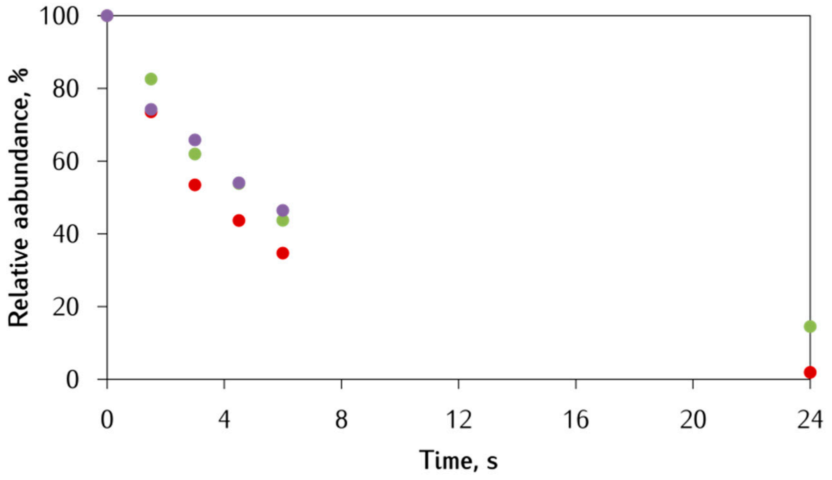

Figure S5. RP-HPLC kinetic traces of DA (200 μM) oxidation in Mops buffer pH 7.0 and 37 °C (blue) in the presence of 100 μM of Fe^{II} (red) and 2 equiv. of peptide (Ac-$\alpha S_{119-132}$: green, Ac-$\alpha S^{P}S_{119-132}$: purple).

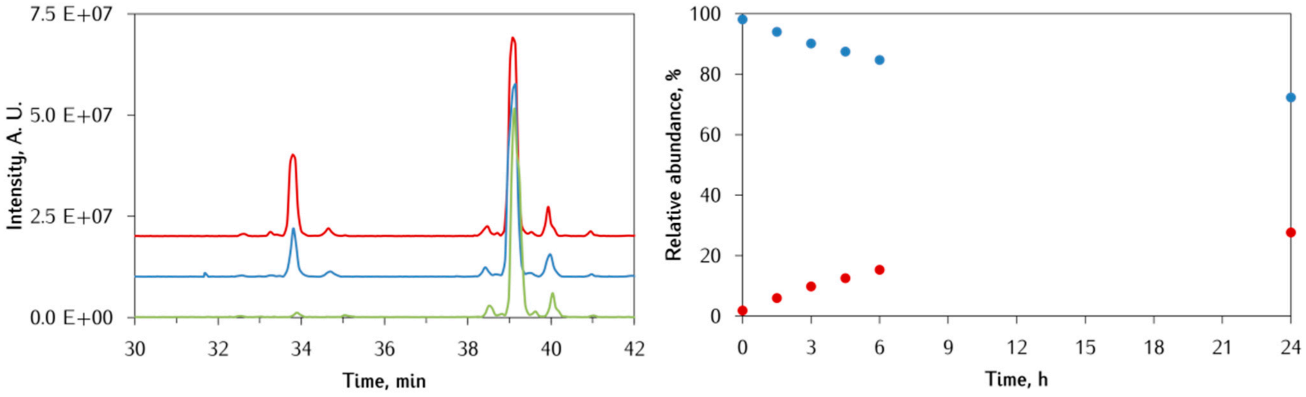

(a) (b)

Figure S6. (a) HPLC-MS elution profiles of Ac-$\alpha S_{119-132}$ (20 μM) in Mops buffer pH 7.0 at 37 °C, in the presence of Fe^{II} (10 μM) and DA (200 μM) at the beginning (blue), and after 6 h (red) and 24 h of incubation (green). (b) Percent modification detected by the HPLC-MS analysis: Ac-$\alpha S_{119-132}$ (t_r 39 min, blue), Ac-$\alpha S_{119-132}$ +16 (t_r 33.8 min, red).

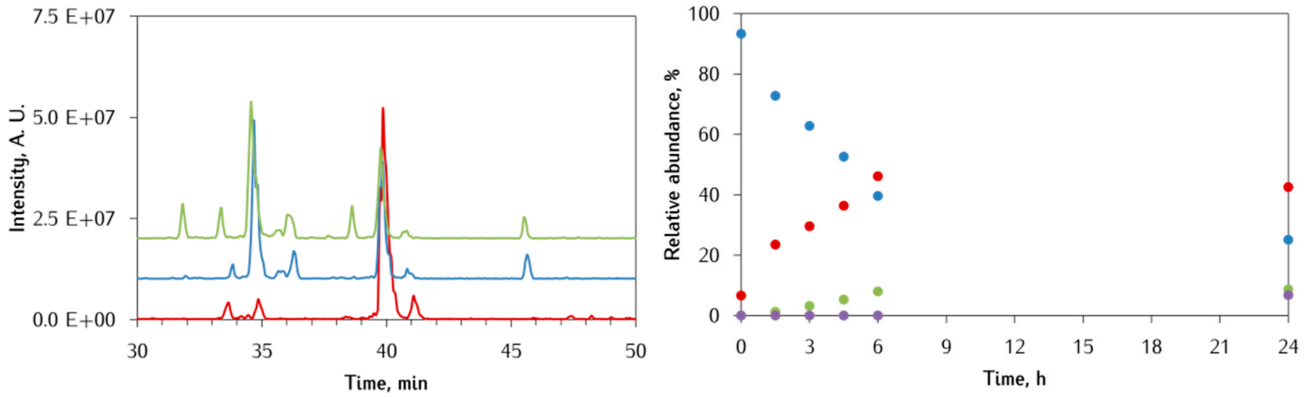

(a) (b)

Figure S7. (a) HPLC-MS elution profiles of Ac-$\alpha SpS_{119-132}$ (20 μM) in Mops buffer pH 7.0 at 37 °C, in the presence of Fe^{II} (10 μM) and DA (200 μM) at the beginning (blue), and after 6 h (red) and 24 h of incubation (green). (b) percent modification detected by HPLC-MS analysis: Ac-$\alpha S^{p}S_{119-132}$ (t_r 40 min, blue), Ac-$\alpha S^{p}S_{119-132}$ +16 (t_r 34.6 min, red), Ac-$\alpha S^{p}S_{119-132}$ +32 (t_r 36.4 min, green), Ac-^{119}DPDNEAYEM*^{127} fragment (t_r 32 min, purple).

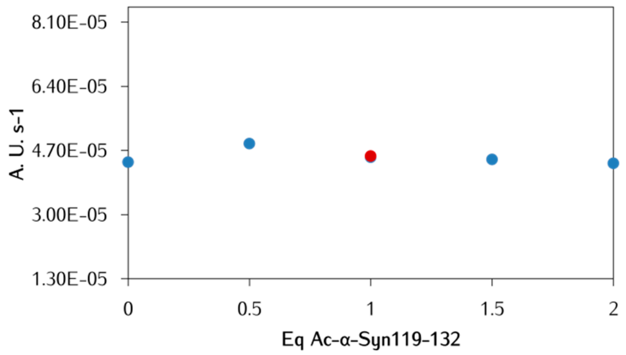

Figure S8. Slope of the kinetic traces of 3 mM DA oxidation by 25 μM Cu^{II} and 0-2 eq of Ac-$\alpha S_{119\text{-}132}$ in Hepes buffer pH 7.4, 37 °C. Red circle refers to DA autoxidation, while blue ones to Cu^{II}-Ac-$\alpha S_{119\text{-}132}$.
